# Supplementary material for: Imprinting Electrically Switchable Scalar Spin Chirality by Anisotropic Strain in a Kagome Antiferromagnet
Source: Adv Sci (Weinh). 2025 Jun 10;12(29):e02569. doi: 10.1002/advs.202502569 (PMC12362762; doi:10.1002/advs.202502569)
Supplement: Supplementary file 1 — Supporting Information [file ADVS-12-e02569-s001.pdf]

ADVANCED SCIENCE
Open Access

Supporting Information

for *Adv. Sci.*, DOI 10.1002/advs.202502569

Imprinting Electrically Switchable Scalar Spin Chirality by Anisotropic Strain in a Kagome Antiferromagnet

*Debjoty Paul, Shivesh Yadav, Shikhar Gupta, Bikash Patra, Nilesh Kulkarni, Debashis Mondal, Kaushal Gavankar, Sourav K. Sahu, Biswarup Satpati, Bahadur Singh, Owen Benton and Shouvik Chatterjee**

Supplementary Information: Imprinting electrically switchable scalar spin chirality by anisotropic strain in a Kagome antiferromagnet

Debjoty Paul,[1,*] Shivesh Yadav,[1,*] Shikhar Gupta,[1] Bikash Patra,[1] Nilesh Kulkarni,[1] Debashis Mondal,[1] Kaushal Gavankar,[1] Sourav K. Sahu,[1,2] Biswarup Satpati,[3] Bahadur Singh,[1] Owen Benton,[4] and Shouvik Chatterjee[1,†]

[1]*Department of Condensed Matter Physics and Materials Science, Tata Institute of Fundamental Research, Homi Bhabha Road, Mumbai 400005, India*
[2]*School of Physical Sciences, National Institute of Science Education and Research, An OCC of Homi Bhabha National Institute, Jatni 752050, India*
[3]*Surface Physics & Material Science Division, Saha Institute of Nuclear Physics, A CI of Homi Bhabha National Institute, 1/AF Bidhannagar, Kolkata 700064, India*
[4]*School of Physical and Chemical Sciences, Queen Mary University of London, London E1 4NS, United Kingdom*

S1: Quasi-epitaxial growth of α-tantalum on c-plane sapphire substrate

An 11 nm thick tantalum (Ta) layer is synthesized as a buffer layer on top of an epitaxial grade c-plane sapphire substrate using RF magnetron sputtering. Ta(110) planes grow in the out-of-plane direction as shown in Fig. S1a. The 2-fold symmetric Ta(110) planes have three different equally probable orientations when synthesized on 6-fold symmetric c-plane sapphire substrates, which are separated by an angle of 120° from each other, as shown in Fig. S1c,d. The quasi-epitaxial growth of Ta, shown in Fig. S1b, provides a quasi-six-fold symmetric surface for the epitaxial integration of $Mn_3Sn(0001)$ atomic layers, but with an anisotropic strain applied in the in-plane directions, as discussed in the main text. The Laue oscillations in the $\theta - 2\theta$ x-ray diffraction (XRD) scan in Fig. S1 establish that Ta atomic layers grow smoothly on c-plane sapphire. The atomic force microscopy (AFM) scan of a 90 nm thick Mn_3Sn film, shown in Fig. S2a, establishes that the thin film heterostructures have a smooth surface with an RMS roughness of $\approx$ 1.3 nm over a 5 $\times$ 5 μm^2 field of view. Rutherford back-scattering spectrometry (RBS) was used to determine the composition of Mn_3Sn thin films, shown in Fig. S2b, which establishes the stoichiometric ratio of 3:1 for Mn:Sn in Mn_3Sn/Ta heterostructures. A high-resolution transmission electron microscopy (HR-TEM) image is shown in Fig. S2c, where the (0001) and $(11\bar{2}0)$ planes are identified. The estimated d-spacings of the planes are similar to what has been estimated from the XRD measurements, described in the main text and in section S2.

S2: Anisotropic strain in Mn_3Sn atomic layers and their incorporation in numerical calculations

In order to estimate the anisotropic strain in Mn_3Sn thin film heterostructures, reciprocal space maps (RSM) around the $\{20\bar{2}1\}$ family of plane viz. $(20\bar{2}1)$, $(02\bar{2}1)$ & $(2\bar{2}01)$ and for (0002) were obtained as shown in Fig. 1 in the main text and Fig. S3. For each of the RSMs around the asymmetric Bragg peak, the corresponding RSM for the (0002) peak at the same ϕ value was also obtained. A two-dimensional Gaussian fit of the RSM plots (2θ, ω) was performed to estimate both the mean value and the standard deviation from the mean of the diffraction spots of the $\{20\bar{2}1\}$ family of planes. The conversion from the (2θ, ω) co-ordinates to ($q_{\parallel}$, $q_{\perp}$) follows as:

$$q_{\parallel} = \frac{1}{\lambda}\left[Sin(\omega) + Sin(2\theta - \omega)\right] \tag{S1}$$

$$q_{\perp} = \frac{1}{\lambda}\left[Cos(2\theta - \omega) - Cos(\omega)\right] \tag{S2}$$

The reciprocal lattice vectors ($q_{\parallel}$, $q_{\perp}$) were then used to estimate the lattice parameters.

$$d_{hkl} = \frac{1}{|\hat{q}|}, |\hat{q}| = \sqrt{q_{\parallel}^2 + q_{\perp}^2} \tag{S3}$$

---

*These authors contributed equally to this work
†Authors to whom correspondence should be addressed: shouvik.chatterjee@tifr.res.in

$$\frac{1}{d_{hkl}} = \frac{4}{3}\left[\frac{(h^2+hk+k^2)}{a^2}\right] + \frac{l^2}{c^2} \tag{S4}$$

Due to the spread observed in the RSM plots, we have used the mean values of $\vec{q}$ and the corresponding bond lengths, which were found to be different for the three measured planes: $(20\bar{2}1)$, $(02\bar{2}1)$ & $(2\bar{2}01)$. The calculated lattice parameters for the Kagome triangles were found to be 2.845Å, 2.860Å, & 2.856Å, as shown in Fig. 1a in the main text, from where the in-plane anisotropic tensile strain in Mn_3Sn was estimated. The out-of-plane lattice parameter c was found to be 4.536Å, slightly compressed from the bulk value [1]. However, the estimated standard deviation quantifying the spread in $\vec{q}$ was found to be similar: 0.005Å^{-1} for all the diffraction spots in $\{20\bar{2}1\}$ family of plane.

The measured in-plane tensile strain and out-of-plane compressive strain were incorporated in the evaluation of the α_{ij} parameters in the Hamiltonian described in eqn. 2 in the main text. The crystal structure of Mn_3Sn consists of a bi-layer stacking of Kagome planes as shown in Fig. S4a. The microscopic spin Hamiltonian for Mn_3Sn can be written as

$$H = \sum_{\langle i,j\rangle} J_{ij}\mathbf{S_i}.\mathbf{S_j} + \sum_{\langle i,j\rangle} \mathbf{D_{ij}}.(\mathbf{S_i}\times\mathbf{S_j})) + \sum_i K(\hat{\mathbf{n}}_\mathbf{i}.\mathbf{S_i})^2 \tag{S5}$$

where we consider only the nearest neighbor interactions, which consist of both the nearest neighbors in the x-y plane and those in the successive x-y planes [2]. There are six Mn spins ($\mathbf{S_i}$) in the unit cell, which form a Kagome triangle in the top layer ($i = 1, 2, 3$) and another in the bottom layer ($i = 1', 2', 3'$), as shown in Fig. S4b. For a particular Mn spin, there are four nearest neighbor exchange interactions, two in-plane and two out-of-plane. For example, for Mn spin at position *1* in the top layer, $\mathbf{S_1}$, the relevant Heisenberg exchange terms are $J_{12}\mathbf{S_1}.\mathbf{S_2} + J_{13}\mathbf{S_1}.\mathbf{S_3} + J_{12'}\mathbf{S_1}.\mathbf{S_{2'}} + J_{13'}\mathbf{S_1}.\mathbf{S_{3'}}$, shown in Fig. S4b. However, inversion symmetry keeps the Hamiltonian invariant under the transformation $\mathbf{S_i} \leftrightarrow \mathbf{S_{i'}}, \forall i = 1, 2, 3$. Therefore,

$$J_{12}\mathbf{S_1}.\mathbf{S_2} + J_{13}\mathbf{S_1}.\mathbf{S_3} + J_{12'}\mathbf{S_1}.\mathbf{S_{2'}} + J_{13'}\mathbf{S_1}.\mathbf{S_{3'}} \equiv (J_{12} + J_{12'})\mathbf{S_1}.\mathbf{S_2} + (J_{13} + J_{13'})\mathbf{S_1}.\mathbf{S_3} \tag{S6}$$

Therefore, eqn. S3 can be recast into eqn. 1 in the main text, where only in-plane exchange interactions are considered.

For simplicity, we have assumed that the λ parameter, as defined in the main text, remains the same for both in-plane and the corresponding out-of-plane interactions, i.e. for example, it remains the same for both J_{12} and $J_{12'}$ and so on. Therefore, for the estimation of α_{ij}, we have averaged the relative changes in length, $(l_{ij} - l_0)/l_0$, over in-and out-of-plane bonds, where l_{ij} and l_0 are defined in the main text.

S3: Strain Analysis from HR-TEM

High-resolution transmission electron microscopy (HR-TEM) was employed to investigate the local strain distribution in the Ta/Mn_3Sn heterostructures. The acquired TEM image, shown in Fig. S5a was analyzed using the open-source program Strain++[3], implementing geometric phase analysis (GPA), as detailed in ref. [4], to extract quantitative strain maps, shown in Fig. S5b. The strain maps reveal significant local strain up to ≈ 8% and strain gradients arising from the dislocations in the bulk of the sample. Significant dislocation density in Mn_3Sn atomic layers arises due to large lattice and symmetry mismatch between Mn_3Sn(0001) and Ta(110) surfaces (please see Figs. S1c and S6a). These local strain fields result in local inversion symmetry breaking in Mn_3Sn, giving rise to bulk in-plane DM interaction in Mn_3Sn/Ta heterostructures. As explained in the main text, in-plane DM interaction plays a crucial role in the emergence of scalar spin chirality and corresponding Berry phase induced anomalous Hall conductivity in the Kagome plane in Mn_3Sn/Ta heterostructures.

S4: Ab-initio simulations of the Mn_3Sn/Ta heterointerface

We have constructed a heterostructure consisting of Ta(110) and Mn_3Sn(0001) surface. The heterostructure is formed by combining 1×3 supercell of a four-layer Ta(110) surface with 1×$\sqrt{3}$ orthorhombic cell of a four-layer Mn_3Sn(0001) surface, resulting in a lattice mismatch of about 8%. Structural relaxation induces out-of-plane displacement of the Mn atoms and facilitates the formation of stable bonds between Mn and Ta atoms at the interface, as shown in Fig. S6a. Due to the significant interfacial anisotropic strain, the Mn-Mn atomic distances deviate from their pristine values reducing the C_{3v} symmetry of the Mn Kagome triangles to C_1, and breaks the inversion symmetry

of the pristine Mn_3Sn(right panel of Fig. S6a, also shown in Fig. 1 in the main text), leading to the development of a complex spin canting pattern.

To further understand the electronic properties of the heterostructure, we examine the electrostatic potential profile as a function of the position along the z-direction, as depicted in Fig. S6b. The analysis reveals a built-in potential difference ΔV=1.73 eV between the Ta and Mn_3Sn layers, which drives a charge transfer across the heterointerface. Fig. S6c shows the isosurface of the charge density differences, highlighting electron accumulation at the interface. To further assess the charge transfer, we define the linear charge density along the z direction $\rho(z)$ averaged over the x-y plane, such that $\int \rho(z)dz$ gives the total number of electrons. We have performed the calculations for the entire heterostructure and individual slabs containing only Mn_3Sn and Ta with the same geometry as the heterostructure. Subsequently, we have calculated the difference between the respective linear charge densities using the following formula:

$$\Delta\rho(z) = \rho_{Ta/Mn_3Sn}(z) - \rho_{Ta}(z) - \rho_{Mn_3Sn}(z) \tag{S7}$$

The result of the calculation is shown in Fig. S6d, where regions of charge accumulation (red) and depletion (blue) illustrate the interfacial charge redistribution. This redistribution arises from the hybridization of two materials, indicating significant electronic interaction at the interface.

S5: Pulse measurement setup and the estimation of the effective temperature of the heat-driven switching

The details of the switching experiment setup are provided in the Methods section. For the switching experiments shown in Fig. 4 in the main text and in Fig. S10, a write current pulse was applied following which Hall resistance (R_{xy}) was measured after a time delay. The read current was kept constant at 100 μA. Two different measurement protocols were followed viz. i) type I - in which the pulse width was kept constant at 12 ms and pulse amplitude was varied (Fig. 4c, S10e) and ii) type II - in which pulse amplitude was kept constant at 100 mA and pulse width was varied (Fig. 4d, S10f). ΔR_{xy} was calculated from the change in the Hall resistance before and after the application of the electrical pulse under a bias field applied along Mn_3Sn(0001). The longitudinal resistance R_{xx} of the Hall bar device was monitored during the application of electrical pulse to estimate the transient temperature of the device due to Joule heating by the applied pulse. Typical time scans of longitudinal voltage(V_{xx}) measured during the switching process are shown in Fig. S9a and S10a, where we use the voltage V_{edge} to obtain a conservative estimate of the transient temperature. The mapping from the measured R_{xx} to the transient device temperature in Ta(11 nm) /Mn_3Sn(90 nm) /Ta(11 nm) /AlO_x (shown in Fig. 4 in the main text) and Ta(11 nm) /Mn_3Sn(90 nm) /AlO_x (shown in Fig. S10e,f) is done using the R_{xx} vs T plots shown in Fig. S9b and S10b, respectively. Temperature dependence of longitudinal resistance R_{xx} of the device was measured from 5 K to 390 K and was smoothly extrapolated to higher temperatures in both the cases.

S6: Subtraction of the contribution of the Ta layer in the electrical transport measurement of Mn_3Sn/Ta heterostructures

To estimate the anomalous Hall conductivity (AHC) of Mn_3Sn from our measurements, contributions from the Ta layer need to be subtracted. Two different layer stacks of Mn_3Sn/Ta heterostructures considered in this work are Ta(11 nm) /Mn_3Sn(90 nm) /AlO_x(8 nm) and Ta(11 nm) /Mn_3Sn(90 nm) /Ta(11 nm) /AlO_x(8 nm) synthesized on an insulating c-plane sapphire substrate. Since the substrate is an insulator, a basic shunt model (or the parallel resistor model) of two resistances $R_{xx}^{Mn_3Sn}$ & R_{xx}^{Ta}, participating in the electrical transport measurement was used. An identical device was fabricated from Ta(11 nm) /AlO_x(8 nm) heterostructure synthesized on c-plane sapphire to obtain R_{xx}^{Ta}. Once R_{xx}^{Ta} is known, $R_{xx}^{Mn_3Sn}$ was calculated as

$$\frac{1}{R_{xx}^{Mn_3Sn}} = \left(\frac{1}{R_{xx}^{Total}} - \frac{1}{R_{xx}^{Ta}}\right) \tag{S8}$$

The current through the Mn_3Sn layer in Mn_3Sn/Ta heterostructures is given by,

$$I_{Mn_3Sn} = I_{Total} \times \frac{R_{xx}^{Ta}}{(R_{xx}^{Mn_3Sn} + R_{xx}^{Ta})} \tag{S9}$$

Where I_{Total} and I_{Mn_3Sn} are the total current and the current flowing through the Mn_3Sn layer, respectively.

The Hall resistance of the Mn_3Sn layer i.e., $R_{xy}^{Mn_3Sn}$, was calculated considering the same parallel resistor model of two resistors. The Hall voltages from the Mn_3Sn and Ta layers are denoted as $V_{xy}^{Mn_3Sn}$ and V_{xy}^{Ta}, respectively. In the parallel resistor model, we now have two cells with voltages $V_{xy}^{Mn_3Sn}$ and V_{xy}^{Ta}, with internal resistances $R_{xx}^{Mn_3Sn}$ and R_{xx}^{Ta}, respectively, as shown in Fig. S11, that are connected in parallel to a voltmeter, which measures the total Hall voltage (V_H) from the device. Using Kirchoff's law we obtain,

$$V_{xy}^{Mn_3Sn} = \left\{ \frac{V_H(R_{xx}^{Ta} + R_{xx}^{Mn_3Sn}) - V_{xy}^{Ta} R_{xx}^{Mn_3Sn}}{R_{xx}^{Ta}} \right\} \tag{S10}$$

Where V_H, $V_{xy}^{Mn_3Sn}$ & V_{xy}^{Ta} are the total Hall voltage of the stack, Hall voltage from Mn_3Sn layer, and the Hall voltage from Ta layer, respectively. Furthermore,

$$R_{xx}^{Ta} = \rho_{xx}^{Ta} \times \left(\frac{w}{l \times t_{Ta}} \right) \tag{S11}$$

$$R_{xx}^{Mn_3Sn} = \rho_{xx}^{Mn_3Sn} \times \left(\frac{w}{l \times t_{Mn_3Sn}} \right) \tag{S12}$$

where l & w are the length and width of the device, respectively, and t_{Ta} & t_{Mn_3Sn} are the thicknesses of the Ta and Mn_3Sn atomic layers, respectively. Using eqns. S8 & S9 in eqn. S7 we estimate $V_{xy}^{Mn_3Sn}$ and calculate the Hall resistivity $\rho_{xy}^{Mn_3Sn}$

$$V_{xy}^{Mn_3Sn} = \left\{ \frac{V_H\left(\frac{\rho_{xx}^{Ta}}{t_{Ta}} + \frac{\rho_{xx}^{Mn_3Sn}}{t_{Mn_3Sn}}\right) - V_{xy}^{Ta} \frac{\rho_{xx}^{Mn_3Sn}}{t_{Mn_3Sn}}}{\frac{\rho_{xx}^{Ta}}{t_{Ta}}} \right\} \tag{S13}$$

Finally, longitudinal ($\sigma_{xx}^{Mn_3Sn}$) and Hall conductivities ($\sigma_{xy}^{Mn_3Sn}$) are obtained as follows

$$\sigma_{xy}^{Mn_3Sn} = \frac{\rho_{xy}^{Mn_3Sn}}{\left(\rho_{xy}^{Mn_3Sn}\right)^2 + \left(\rho_{xx}^{Mn_3Sn}\right)^2} \quad \& \quad \sigma_{xx}^{Mn_3Sn} = \frac{\rho_{xx}^{Mn_3Sn}}{\left(\rho_{xy}^{Mn_3Sn}\right)^2 + \left(\rho_{xx}^{Mn_3Sn}\right)^2} \tag{S14}$$

For Ta(11 nm) /Mn_3Sn(90 nm) /Ta(11 nm) /AlO_x(8 nm) heterostructures, the top Ta layer was assumed to be similar to the bottom Ta layer. Hence, in the parallel resistor model, an effective thickness of 22 nm(11 nm + 11 nm) was used for the Ta layer. We also verified our hypothesis by calculating the resistivity of the top Ta layer by subtracting the resistivity of the Ta(bottom layer)/Mn_3Sn layers from the full heterostructure stack by using the resistivity measured in Ta/Mn_3Sn/AlO_x samples. In this exercise, the estimated resistivity of the top Ta layer was found to be very similar to the bottom Ta layer, validating our hypothesis.

[1] N. H. Sung, F. Ronning, J. D. Thompson, E. D. Bauer, Magnetic phase dependence of the anomalous Hall effect in Mn_3Sn single crystals. *Appl. Phys. Lett.* **112**, 132406 (2018)

[2] J. Liu, L. Balents, Anomalous Hall Effect and Topological Defects in Antiferromagnetic Weyl Semimetals: Mn_3Sn/Ge. *Phys. Rev. Lett.* **119**, 087202 (2017)

[3] https://jjppeters.github.io/Strainpp/

[4] M. Hÿtch, E. Snoeck, R. Kilaas, Quantitative measurement of displacement and strain fields from HREM micrographs, *Ultramicroscopy* **74**, 131 (1998)

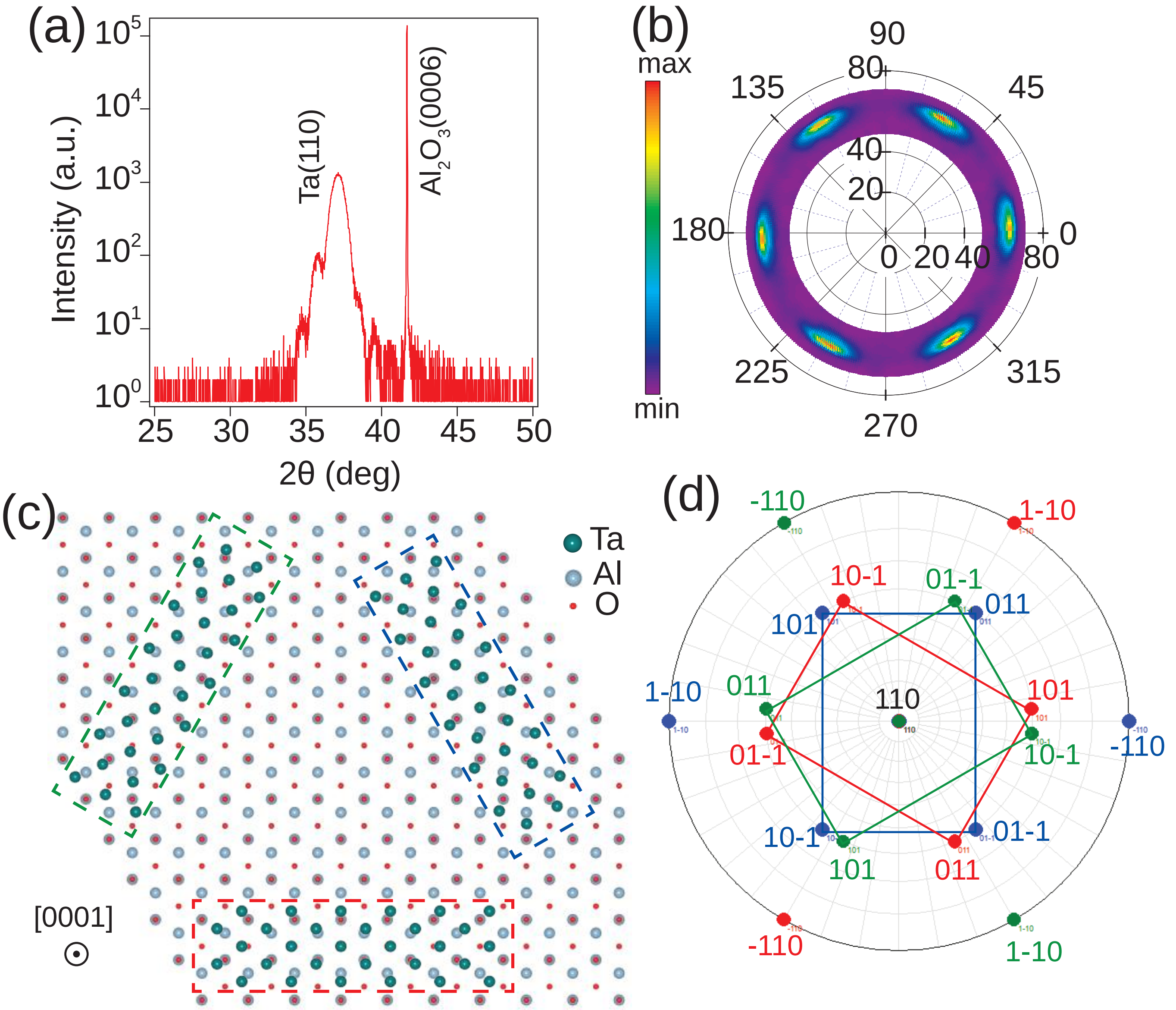

Figure S1: **Structural characterization of Ta layer in Mn_3Sn/Ta heterostructures.** a) Out-of-plane θ-2θ XRD scan of an 11 nm thick Ta thin film synthesized on c-plane sapphire. b) Pole figure plot of Ta{101} family of diffraction peaks showing a six-fold symmetry establishing the epitaxial nature of Ta. c) Simulation of the three equally probable Ta(110) domains on c-plane sapphire. d) Simulation of diffraction pattern showing three equally probable domain orientations of Ta(110) plane giving rise to a quasi-six-fold symmetric epitaxial template as observed in b).

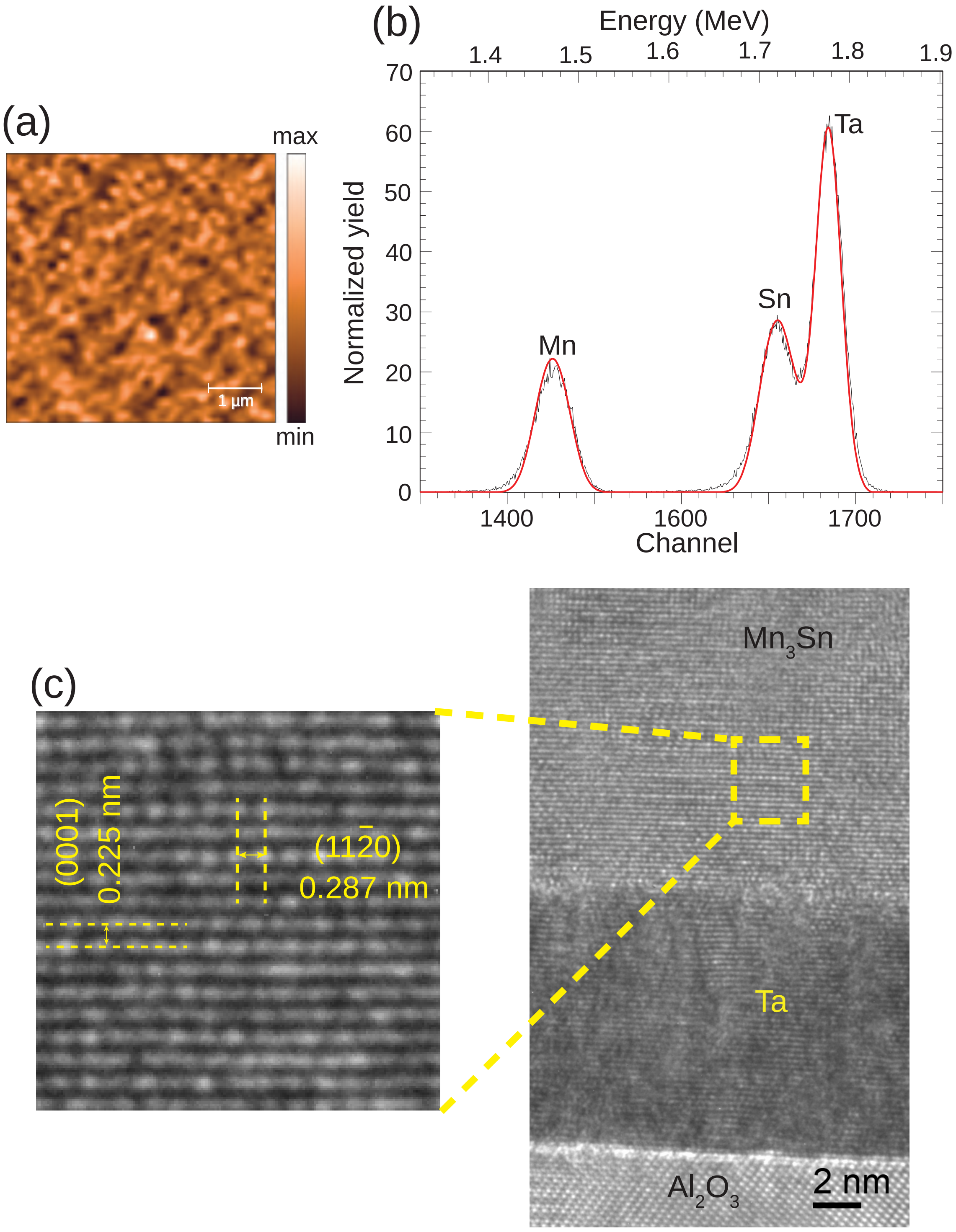

Figure S2: **Characterization of Mn_3Sn/Ta heterostructures.** a) AFM scan of a Mn_3Sn(90 nm)/Ta(11 nm) thin film synthesized on c-plane sapphire. b) RBS data from a Mn_3Sn/Ta thin film heterostructure establishing the Mn:Sn ratio of 3:1 in Mn_3Sn. The corresponding fit is shown in red. c) High-resolution transmission electron microscopy (HR-TEM) image of a Mn_3Sn(90 nm)/Ta(11 nm) thin film heterostructure. The (0001) and (11$\bar{2}$0) planes are highlighted in the zoomed-in image on the left, along with their estimated *d*-spacings.

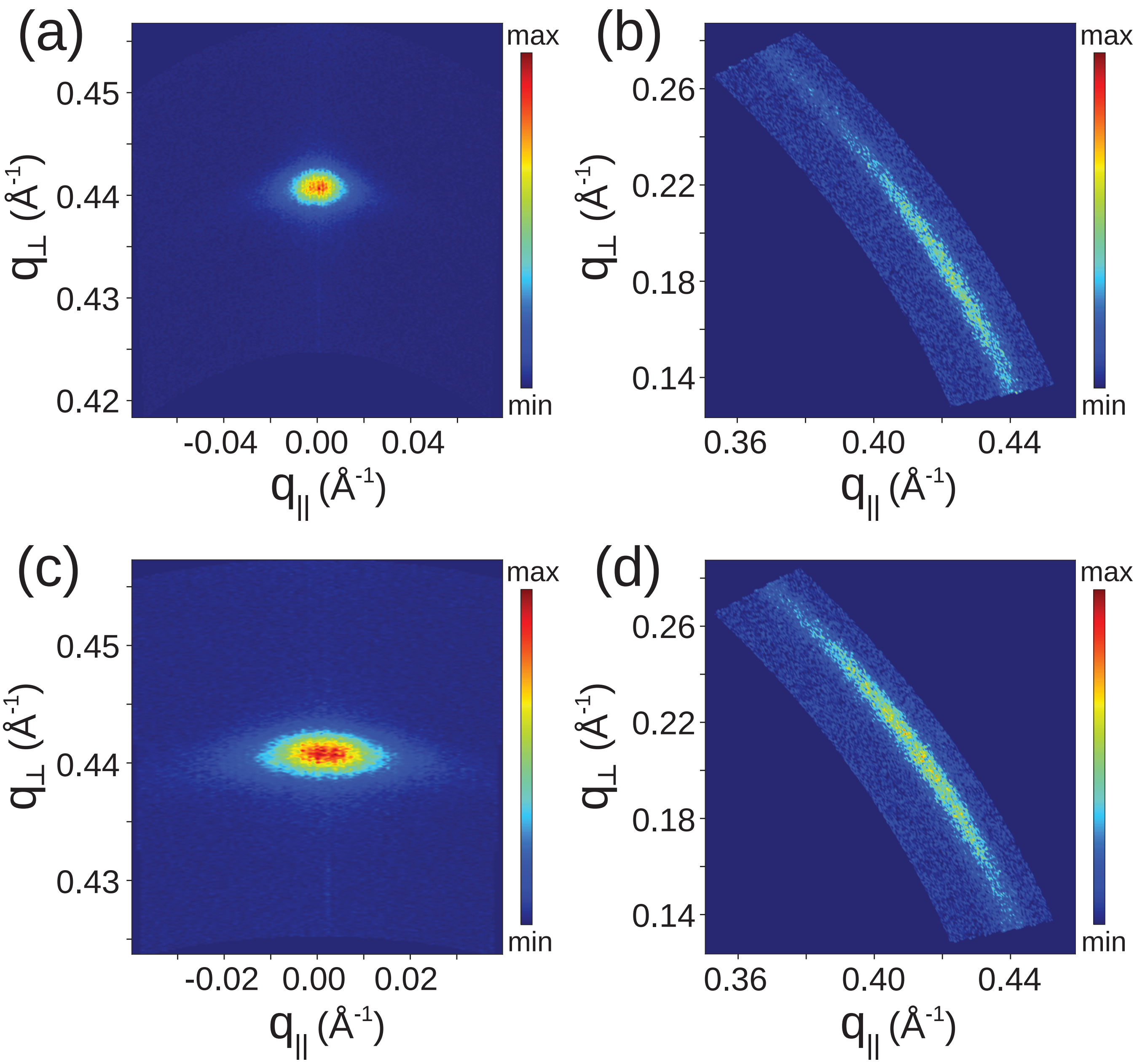

Figure S3: **Reciprocal Space Map (RSM) of the Mn_3Sn $\{20\bar{2}1\}$ family of planes.** RSM of a) (0002), b) $(02\bar{2}1)$, c) (0002) and d) $(2\bar{2}01)$. a) and b) were obtained with the same ϕ value, as were c) and d).

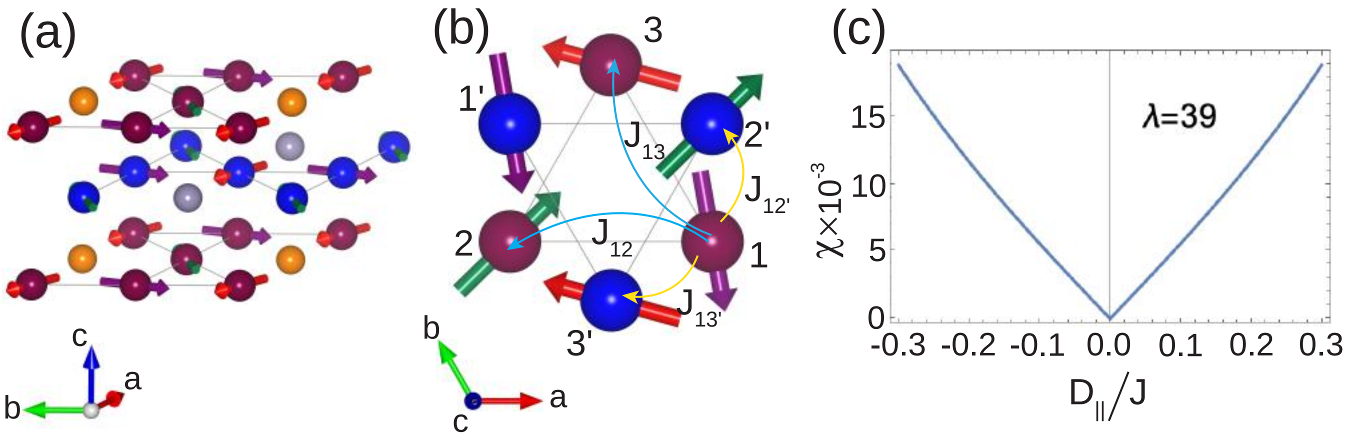

Figure S4: **Non-coplanar spin structure in Mn_3Sn.** a) Three-dimensional crystal structure of Mn_3Sn, which consists of bi-layer stacking of Kagome planes. The manganese (Mn) atoms in the top and bottom layers are shown in red and blue, respectively. b) Nearest-neighbor interactions in Mn_3Sn. For clarity, interactions of only one such Mn spin (Mn spin at position 1 in the top layer) are shown, which consists of two in-plane (J_{12} & J_{13}, shown in blue) and two out-of-plane ($J_{12'}$ & $J_{13'}$, shown in yellow) interactions. c) Calculated scalar spin chirality (χ) as a function of $D_{\|}/J$ for $\lambda = 39$. Note that $\chi = 0$ when $D_{\|} = 0$, and acquires a non-zero value for both $D_{\|} > 0$ and $D_{\|} < 0$.

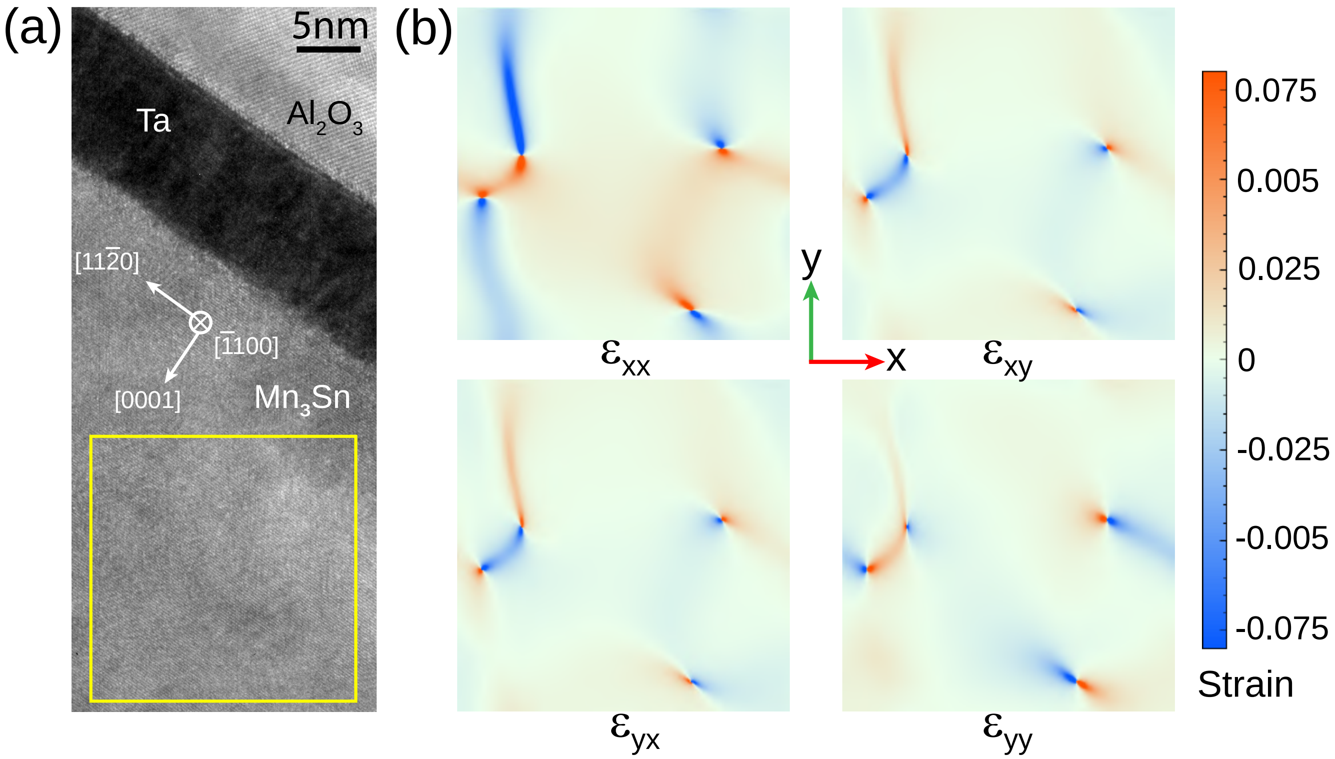

Figure S5: **Strain fields in the Mn_3Sn layer in Mn_3Sn/Ta heterostructure.** a) HR-TEM image of the Ta/Mn_3Sn/Ta sample, showing the area of Mn_3Sn layer used for strain-analysis (yellow square). b) Strain components in Mn_3Sn atomic layer showing large local strain and strain gradient originating from atomic dislocations.

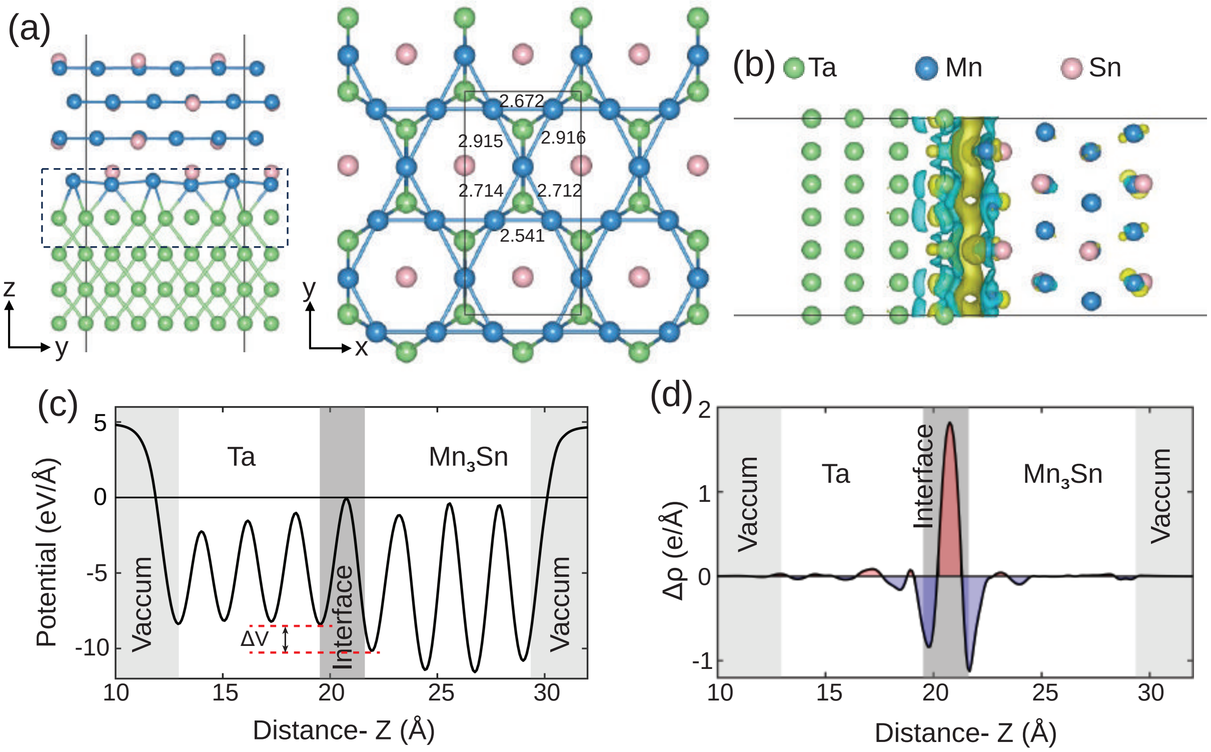

Figure S6: **First-principle calculation of the Ta(110)/Mn_3Sn(0001) heterostructure** a) Side view of the relaxed structure of Ta(110)/Mn_3Sn(0001) heterostructure (left) and a top view of the highlighted rectangular region (right), revealing a reduction of the C_{3v} symmetry of the Kagome triangles to C_1 as well as lifting of the inversion symmetry of the pristine Mn_3Sn crystal structure. b) Electrostatic potential distribution along the z-axis, showing distinct potential profiles for Ta, Mn_3Sn, and the interface. c) Iso-surface of the charge density difference in real space. Yellow and blue iso-surfaces indicate electron accumulation and depletion, respectively. d) Planar-averaged charge density difference along the stacking direction. Charge transfer between Ta and Mn_3Sn leads to interface formation.

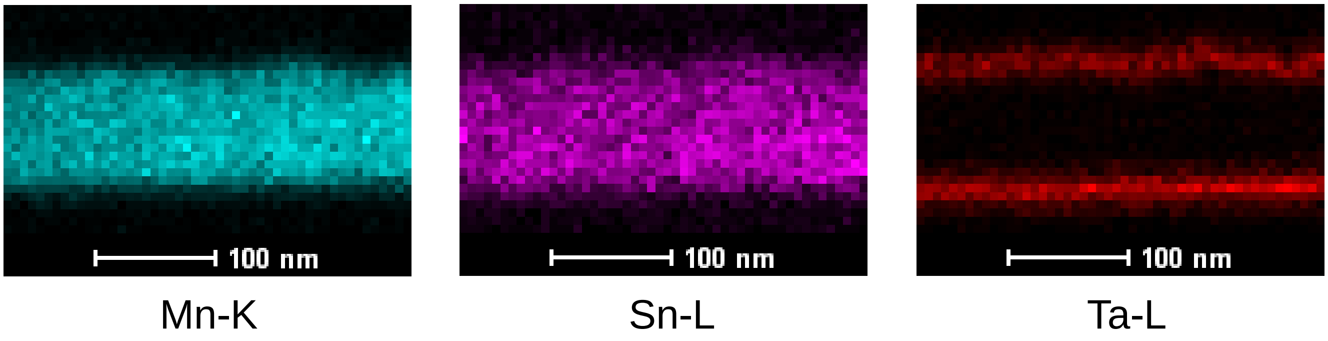

Figure S7: Energy-dispersive x-ray spectroscopy (EDS) data for Mn-K, Sn-L & Ta-L edges in a Ta/Mn_3Sn/Ta/AlO_x heterostructure establishing negligible intermixing at the heterointerfaces.

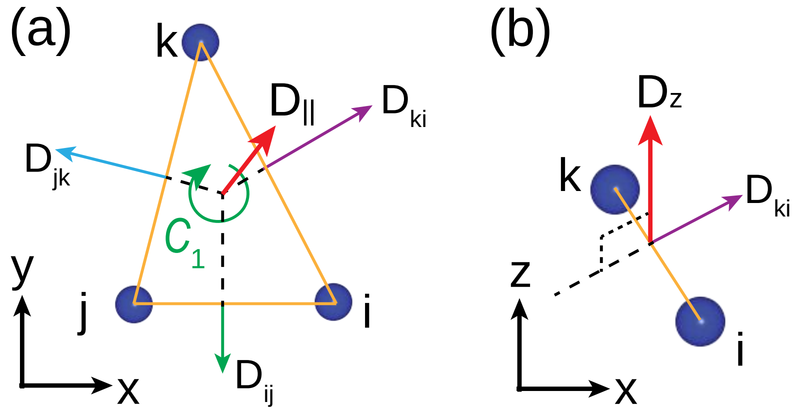

Figure S8: Schematic diagram of the a) in-plane ($D_{||}$) and b) out-of-plane (D_z) components of the Dzyaloshinskii-Moriya (DM) vector in a distorted Kagome triangle.

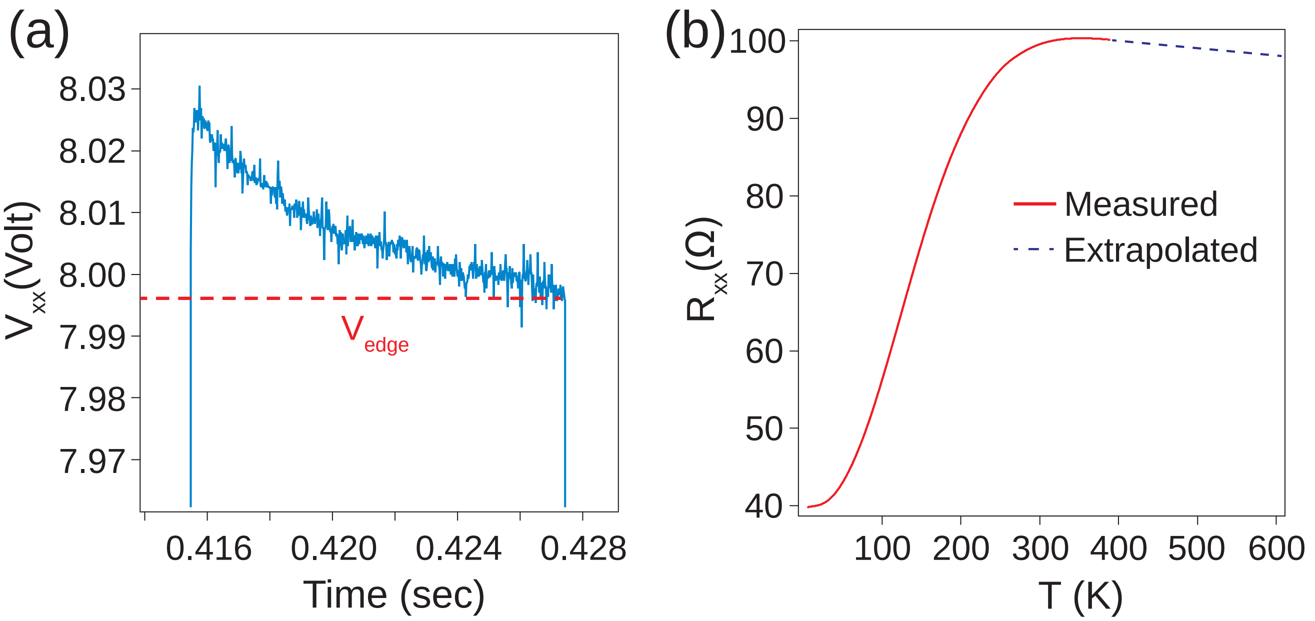

Figure S9: **Estimation of transient device temperature during application of electrical pulse in Ta/Mn_3Sn/Ta/AlO_x heterostructures.** a) The longitudinal voltage V_{xx} during the application of an 80 mA pulse with a pulse width of 12 ms. V_{edge}, which is used for estimating device temperature, is shown in a red dotted line. b) The temperature dependence of longitudinal resistance (R_{xx}) from 5 K to 390 K is shown in red. The measured data is smoothly extrapolated to higher temperatures, shown in a blue dotted line.

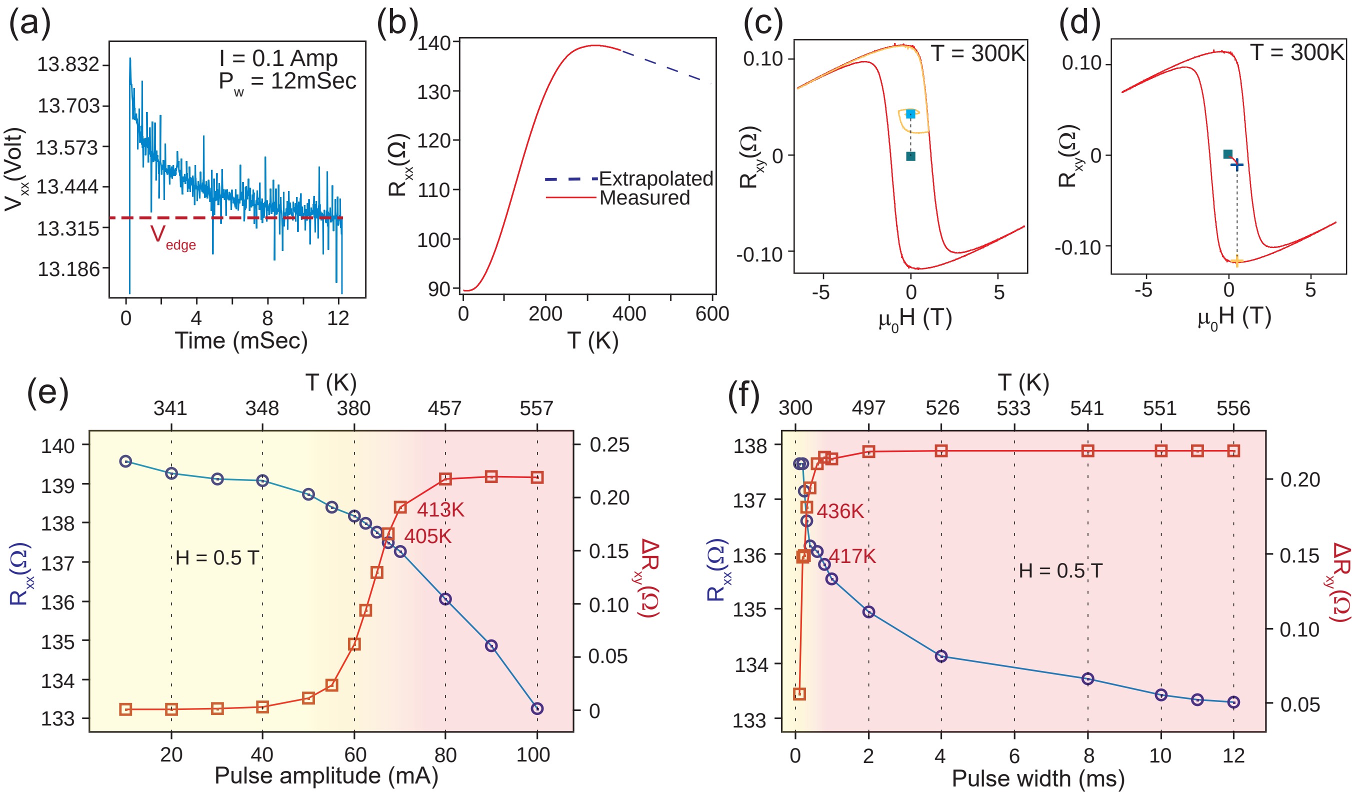

Figure S10: **Thermal assisted switching in Ta/Mn_3Sn/AlO_x heterostructures.** a) The longitudinal voltage V_{xx} during the pulse application. V_{edge}, which is used for estimating device temperature, is shown in a red dotted line. b) The temperature dependence of longitudinal resistance (R_{xx}) from 5 K to 390 K is shown in red. The measured data is smoothly extrapolated to higher temperatures, shown in a blue dotted line. Switching of Anomalous Hall resistance at c) zero field and d) at a bias field of 0.5 T. Switching behavior of AHR (R_{xy}) as a function of e) pulse amplitude, keeping the pulse-width constant at 12 ms, f) pulse width, keeping the pulse amplitude constant at 100 mA. A bias field of 0.5 T cooling field is applied in both cases. For the temperature mapping in e) & f) transient temperature of the device is estimated by measuring the transient V_{xx} during the pulse application as shown in a).

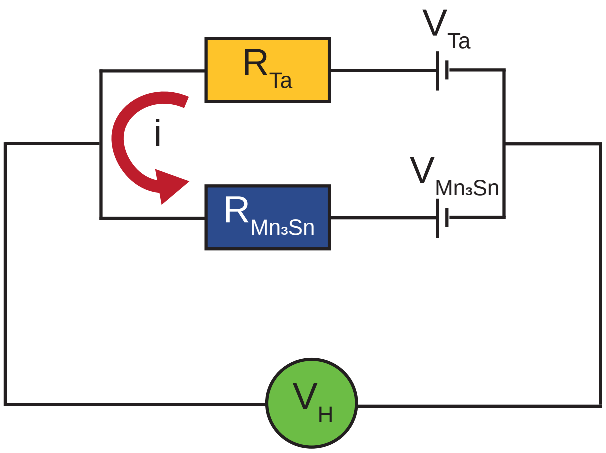

Figure S11: **Parallel cell and Shunt model of the Ta & Mn_3Sn layers in Mn_3Sn/Ta heterostructure.**

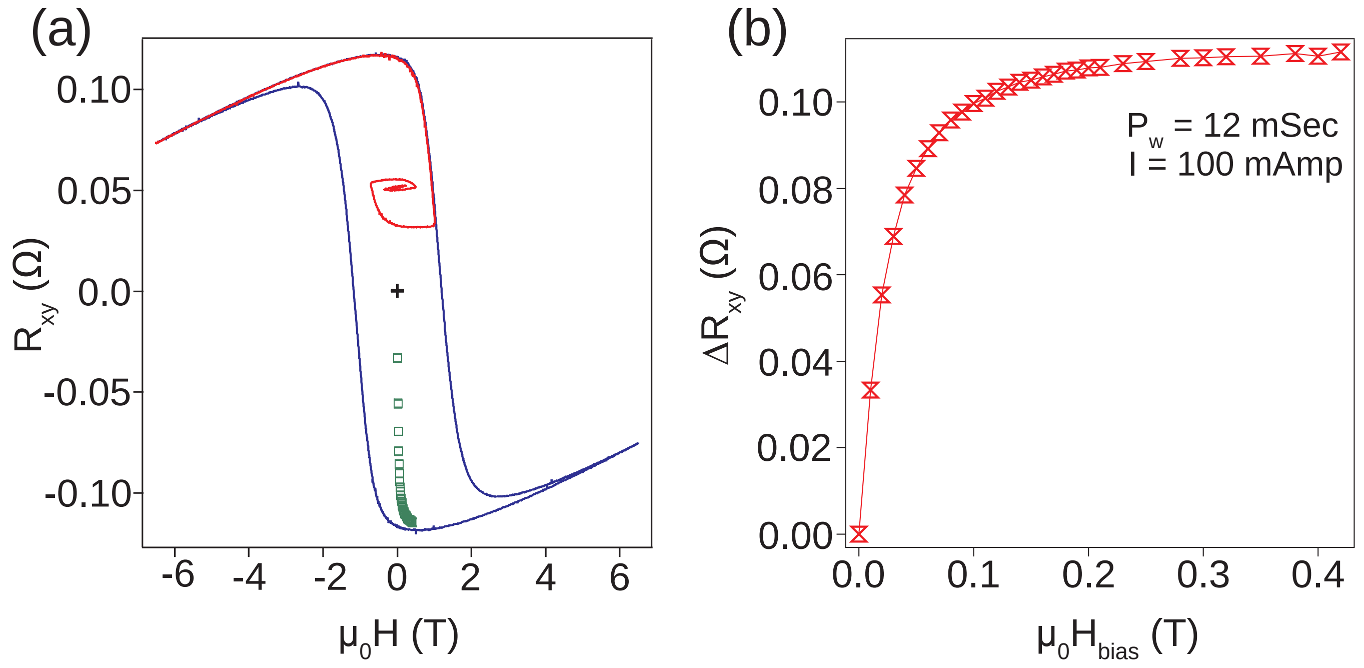

Figure S12: **Multi-state switching of anomalous Hall resistance in a Ta/Mn_3Sn/AlO_x heterostructure.** a) Anomalous Hall resistance states after application of different bias fields. At first, the field is oscillated to zero to ensure there is no trapped field. Application of an electric pulse brings the resistance to 0. Following which application of an electric pulse under different bias fields allows access to the different anomalous Hall resistance states. The anomalous Hall hysteresis loop as a function of applied magnetic field is shown in blue for reference. b) The change in anomalous Hall resistance as a function of applied bias field. In all cases, a single 100 mA pulse of duration 12 ms has been applied.
